# Overexpression of Prothymosin Alpha Predicts Poor Disease Outcome in Head and Neck Cancer

**DOI:** 10.1371/journal.pone.0019213

**Published:** 2011-05-05

**Authors:** Satyendra Chandra Tripathi, Ajay Matta, Jatinder Kaur, Jorg Grigull, Shyam Singh Chauhan, Alok Thakar, Nootan Kumar Shukla, Ritu Duggal, Ajoy Roy Choudhary, Siddhartha DattaGupta, Mehar Chand Sharma, Ranju Ralhan, K. W. Michael Siu

**Affiliations:** 1 Department of Biochemistry, All India Institute of Medical Sciences, New Delhi, India; 2 Department of Chemistry and Centre for Research in Mass Spectrometry, York University, Toronto, Ontario, Canada; 3 Department of Mathematics and Statistics, York University, Toronto, Ontario, Canada; 4 Department of Otorhinolaryngology, All India Institute of Medical Sciences, New Delhi, India; 5 Department of Surgery, Dr. B. R. A. Institute Rotary Cancer Hospital, All India Institute of Medical Sciences, New Delhi, India; 6 Centre for Dental Education and Research, All India Institute of Medical Sciences, New Delhi, India; 7 Department of Pathology, All India Institute of Medical Sciences, New Delhi, India; 8 Joseph and Mildred Sonshine Family Centre for Head and Neck Diseases and Department of Otolaryngology – Head and Neck Surgery, Mount Sinai Hospital, Toronto, Ontario, Canada; 9 Alex and Simona Shnaider Laboratory in Molecular Oncology and Department of Pathology and Laboratory Medicine, Mount Sinai Hospital, Toronto, Ontario, Canada; 10 Department of Otolaryngology – Head and Neck Surgery, University of Toronto, Toronto, Ontario, Canada; Enzo Life Sciences, Inc., United States of America

## Abstract

**Background:**

In our recent study, tissue proteomic analysis of oral pre-malignant lesions (OPLs) and normal oral mucosa led to the identification of a panel of biomarkers, including prothymosin alpha (PTMA), to distinguish OPLs from histologically normal oral tissues. This study aimed to determine the clinical significance of PTMA overexpression in oral squamous cell hyperplasia, dysplasia and head and neck squamous cell carcinoma (HNSCC).

**Methodology:**

Immunohistochemistry of PTMA protein was performed in HNSCCs (n = 100), squamous cell hyperplasia (n = 116), dysplasia (n = 50) and histologically normal oral tissues (n = 100). Statistical analysis was carried out to determine the association of PTMA overexpression with clinicopathological parameters and disease prognosis over 7 years for HNSCC patients.

**Results:**

Our immunohistochemical analysis demonstrated significant overexpression of nuclear PTMA in squamous cell hyperplasia (63.8%), dysplasia (50%) and HNSCC (61%) in comparison with oral normal mucosa (p_trend_<0.001). Chi-square analysis showed significant association of nuclear PTMA with advanced tumor stages (III+IV). Kaplan Meier survival analysis indicated reduced disease free survival (DFS) in HNSCC patients (p<0.001; median survival 11 months). Notably, Cox-multivariate analysis revealed nuclear PTMA as an independent predictor of poor prognosis of HNSCC patients (p<0.001, Hazard's ratio, HR = 5.2, 95% CI = 2.3–11.8) in comparison with the histological grade, T-stage, nodal status and tumor stage.

**Conclusions:**

Nuclear PTMA may serve as prognostic marker in HNSCC to determine the subset of patients that are likely to show recurrence of the disease.

## Introduction

Human prothymosin alpha (PTMA) is a 12.5 kDa, acid-rich, non-histone nuclear protein, comprising of 110 amino acids [1]. PTMA is known to play an important role in cell cycle regulation, proliferation, transcription, chromatin remodeling, oxidative stress-response and apoptosis [2]–[8]. Overexpression of PTMA has been reported in several malignancies such as breast cancer [5], hepatocarcinoma [9], lung cancer [10], neuroblastoma [11], bladder cancer [12], gastric [13] and upper urinary tract transitional cell carcinoma [14]. In addition, PTMA also served as prognostic marker for cancers of the breast, gastric, prostate and bladder [5], [13], [15], [16]. Recently, we identified PTMA among the panel of biomarkers which may find their utility in distinguishing OPLs and HNSCCs from non-malignant tissues, using isobaric tags for relative and absolute quantitation (iTRAQ) labeling and multidimensional liquid chromatography/tandem mass spectrometry (LC-MS/MS) [17].

HNSCC is among the ten most prevalent cancers in the world, with a higher proportion occurring in developing countries. Tobacco, betel quid and alcohol consumption as well as human papilloma virus (HPV) are the major risk factors associated with development of head and neck cancer [18]–[24]. Oral squamous cell carcinoma (OSCC), one of the major subtypes of HNSCC, is often preceded by clinically well-defined lesions, such as leukoplakia, which is causally linked with chronic exposure of the oral mucosa to carcinogens/growth promoters in tobacco and/or betel quid. On an average about 1–2% of these oral lesions transform into cancer annually [25]–[27]. Moreover, more than 50% of all HNSCC patients have advanced disease at the time of diagnosis [28]. Although, there have been advancements in treatment modalities that improve the quality of life and have palliative value, the survival rates for HNSCC patients have not improved markedly (about 50%), often owing to loco-regional recurrence or second primary tumors observed commonly in the survivors [27]–[30]. At present, the most important prognostic factors for HNSCC are histological tumor grade, stage, depth of tumor invasion and involvement of regional lymph nodes at the time of diagnosis. However, none of these factors can predict prognosis of HNSCC effectively, thus emphasizing the importance of identifying novel biomarkers for early detection, risk assessment and accurate prediction of recurrence for this malignancy.

In this study, we aimed to determine the clinical significance of PTMA overexpression in head and neck tumorigenesis. We analyzed the expression of PTMA in a large cohort of clinical samples including normal oral mucosa, squamous cell hyperplasia, dysplasia and HNSCC by immunohistochemistry. Further, we investigated correlations of nuclear PTMA expression with clinicopathological parameters of squamous cell hyperplasia, dysplasia and HNSCC to determine its utility as a biomarker.

## Results

### Correlation of nuclear PTMA in squamous cell hyperplasia, dysplasia and HNSCC with clinicopathological characteristics

**Figure 1:**
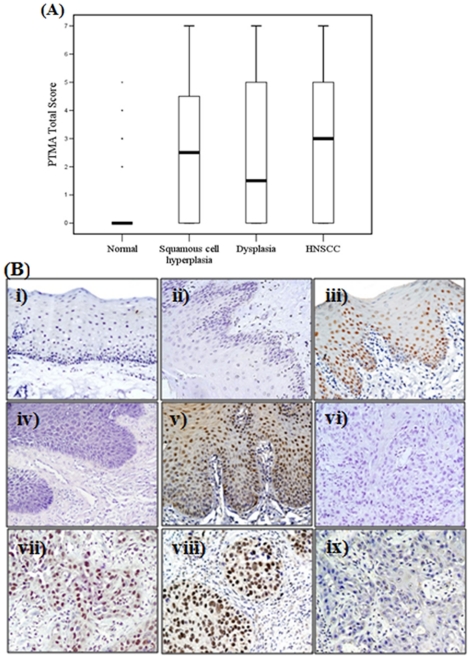
(**a**) **Box-Plot analysis:** Box plots showing distribution of total scores based on immunohistochemistry of PTMA protein in paraffin-embedded sections of oral normal tissues, squamous cell hyperplasia, dysplasia and HNSCC. The vertical axis shows total immunostaining score, obtained as described in the Methods section. Figure shows total score distribution of nuclear PTMA expression in squamous cell hyperplasia (score range 0–7), dysplasia (score range 0–7) and HNSCC (score range 0–7). (**b**) **Immunohistochemical analysis of PTMA in head and neck tissues:** Paraffin-embedded sections of histologically oral normal mucosa, squamous cell hyperplasia, dysplasia and HNSCC were stained using anti-PTMA polyclonal antibody as described in the Methods section. (i) normal oral mucosa showing no PTMA immunostaining; (ii) squamous cell hyperplasia showing no immunostaining for PTMA; (iii) squamous cell hyperplasia showing nuclear PTMA immunostaining in epithelial cells; (iv) dysplasia showing no nuclear PTMA immunostaining; (v) dysplasia depicting nuclear PTMA immunostaining in epithelial cells; (vi) HNSCC section showing no nuclear PTMA staining; (vii) HNSCC section illustrating nuclear PTMA staining in tumor cells; (viii) bladder cancer tissue section showing nuclear PTMA immunostaining; (ix) HNSCC section used as a negative control, showing no PTMA immunostaining in tumor cells; (i–ix original magnification ×200).

To determine the clinical significance of PTMA protein in head-and-neck tumorigenesis, its expression was analyzed in normal oral mucosa, squamous cell hyperplasia, dysplasia and HNSCC using immunohistochemistry. Figure 1A shows the total score distribution of nuclear PTMA in normal oral mucosa, squamous cell hyperplasia, dysplasia and HNSCC. Of the 100 histologically normal tissues analyzed, 88 tissues (88%) showed no detectable PTMA immunostaining in nuclei of epithelial cells in normal tissues (Table 1, Figure 1B (i)). PTMA expression was observed in 5 of 43 (11.6%) paired normal tissues and 7 of 57 (12.3%) unpaired normal tissues only. Chi square trend analysis also showed significant increase in nuclear expression of PTMA in tissues obtained from different stages of head-and-neck tumorigenesis (squamous cell hyperplasia, dysplasia and HNSCC, p_trend_<0.001). Among squamous cell hyperplasia (n = 116), 74 cases (63.8%) showed significant increase in nuclear PTMA immunoreactivity (p<0.001, OR = 12.9, 95% CI = 6.3–26.3) in comparison with normal oral tissues (Table 2 and Figure 1B (iii)). Increased nuclear expression of PTMA was also observed in 50% dysplasia (25 of 50 cases, p<0.001, OR = 7.3, 95% CI = 3.2–16.6) as compared to normal oral tissues (Table 3 and Figure 1B (v)). Tables 1, 2 and 3 summarize correlations of nuclear PTMA with clinicopathological parameters of normal oral mucosa, squamous cell hyperplasia and dysplasia respectively. Interestingly, nuclear PTMA showed significant association with tobacco consumption habits of patients with squamous cell hyperplasia (p<0.001).

**Table 1:** Analysis of PTMA protein expression in Normal oral mucosa and correlation with clinicopathological parameters.

Clinicopathological Features	Total Cases	Nuclear Positive N (%)	Nuclear Negative N (%)	p-value	OR (95% CI)
**Normal oral mucosa**	100	12 (12)	88 (88)		
**Paired**	43	5 (11.6)	38 (88.4)	**------**	**------**
**Unpaired**	57	7 (12.3)	50 (87.7)		
**Age (Median, 47 yrs) (16–71 yrs)**					
<47	67	9 (13.4)	58 (86.6)	0.53	**------**
≥47	33	3 (9.1)	30 (90.9)		
**Gender**					
Male	64	8 (12.5)	56 (87.5)	0.89	**------**
Female	36	4 (11.1)	32 (88.9)		
**Site**					
Tongue	27	4 (14.8)	23 (85.2)		
Buccal Mucosa	13	1 (7.7)	12 (92.3)	0.95	**------**
Others (Alveolus, RMT, FOM)	60	7 (11.7)	53 (88.3)		
* **Habits**					
Tobacco non- consumers	34	1 (2.9)	33 (97.1)	0.17	**------**
Tobacco consumers	35	4 (11.4)	31 (88.6)		

*Tobacco consumption habits were available for 69 subjects. RMT, retromolar trigone; FOM, floor of mouth.

**Table 2:** Analysis of PTMA protein expression in squamous cell hyperplasia and correlation with clinicopathological parameters.

Clinicopathological Features	Total Cases	Nuclear Positive N (%)	Nuclear Negative N (%)	p-value	OR (95% CI)
**Squamous Cell Hyperplasia**	116	74 (63.8)	42 (36.2)	**<0.001** *	**12.9 (6.3–26.3)**
**Age (Median, 38 yrs) (16–69 yrs)**					
<38	99	68 (68.7)	31 (31.3)	**0.008**	**0.25 (0.08–0.73)**
≥38	17	6 (35.3)	11 (64.7)		
**Gender**					
Male	87	58 (66.7)	29 (33.3)	0.265	
Female	29	16 (55.2)	13 (44.8)		
**Site**					
Tongue	4	1 (25.0)	3 (75.0)		
Buccal Mucosa	83	56 (67.5)	27 (32.5)	0.019	
Others (Alveolus, RMT, FOM)	29	17 (58.6)	12 (41.4)		
** **Habits**					
Tobacco non- consumers	12	1 (8.3)	11 (91.7)	**<0.001**	**25.9 (3.2–209.3)**
Tobacco consumers	104	73 (70.2)	31 (29.8)		

*Normal vs. squamous cell hyperplasia;

**Tobacco consumption habits include tobacco chewing (betel quid, areca nut or pan masala) and/or smoking of bidi or cigarettes.

**Table 3:** Analysis of PTMA protein expression in dysplasia and correlation with clinicopathological parameters.

Clinicopathological Features	Total Cases	Nuclear Positive N (%)	Nuclear Negative N (%)	p-value	OR (95% CI)
**Dysplasia**	50	25 (50.0)	25 (50.0)	**<0.001** *	**7.3 (3.2–16.6)**
**Age** (Median, 45 yrs) (20–75 yrs)					
<45	22	12 (54.5)	10 (45.5)	0.569	**-----**
≥45	28	13 (46.4)	15 (53.6)		
**Gender**					
Male	43	21 (48.8)	22 (51.2)	0.684	**-----**
Female	7	4 (57.1)	3 (42.9)		
**Site**					
Tongue	7	2 (28.6)	5 (71.4)		
Buccal Mucosa	33	18 (54.5)	15 (45.5)	0.317	**-----**
Others (Alveolus, RMT, FOM)	10	5 (50.0)	5(50.0)		
**Histopathological grade**					
Mild Dysplasia	39	20 (51.3)	19 (48.7)	0.73	**-----**
Moderate Dysplasia	9	4 (44.4)	5 (55.6)		
Severe Dysplasia	2	1 (50.0)	1 (50.0)		
** **Habits**					
Tobacco non- consumers	0	0	0	**-----**	**-----**
Tobacco consumers	50	25 (50.0)	25 (50.0)		

*Normal vs. dysplasia; Squamous cell hyperplasia vs. dysplasia, p = 0.097;

**Tobacco consumption habits include tobacco chewing (betel quid, areca nut or pan masala) and/or smoking of bidi or cigarettes.

Among HNSCC, 61% cases showed nuclear localization of PTMA in tumor cells as compared to normal oral tissues (p<0.001, OR = 11.5, 95% CI = 5.7–23.7; Table 4 and Figure 1B (vii)). The clinicopathological parameters of HNSCC and their correlations with nuclear PTMA expression are shown in Table 4. Interestingly, nuclear PTMA showed significant association with advanced tumor stage (p = 0.03). Notably, none of the HNSCC tissues showed cytoplasmic PTMA immunostaining. The positive control (bladder cancer) showed intense nuclear PTMA expression (Figure 1B (viii)) while no immunostaining was observed in tissue sections used as negative controls where the primary antibody was replaced by isotype specific IgG (Figure 1B (ix)).

**Table 4:** Analysis of PTMA protein expression in HNSCC and correlation with clinicopathological parameters.

Clinicopathological Features	Total Cases	Nuclear Positive N (%)	Nuclear Negative N (%)	p-value	OR (95% CI)
**HNSCC**	100	61 (61.0)	39 (39.0)	**<0.001** *	**11.5 (5.7–23.7)**
**Age** (Median, 53 yrs) (25–85 yrs)					
<53	49	28 (57.1)	21 (42.9)	0.43	-----
≥53	51	33 (64.7)	18 (35.3)		
**Gender**					
Male	75	45 (60.0)	30 (40.0)	0.72	-----
Female	25	16 (64.0)	9 (36.0)		
**Site**					
Tongue	44	26 (59.1)	18 (40.1)		
Buccal Mucosa	31	17 (54.8)	14 (45.2)	-----	-----
Others (Alveolus, Lip, RMT, FOM)	25	18 (72.0)	7 (28.0)		
**Histopathological grade**	100	74 (63.8)	42 (36.2)		
WDSCC	45	25 (55.6)	20 (44.4)	0.55	1.3 (0.6–2.9)
MDSCC	49	32 (65.3)	17 (34.7)		
PDSCC	6	4 (66.7)	2 (33.3)		
**T-Stage**					
T_1_+T_2_	39	21 (53.8)	18 (46.2)	0.24	-----
T_3_+T_4_	61	40 (65.6)	21 (34.4)		
**Nodal Status**					
N_0_	33	16 (48.5)	17 (51.5)	0.07	-----
N_1–3_	67	45 (67.2)	22 (32.8)		
**Tumor Stage**					
I+II	20	8 (40.0)	12 (60.0)	**0.031**	**2.9 (1.1–8.1)**
III+IV	80	53 (66.3)	27 (33.7)		
** **Habits**					
Tobacco non- consumers	22	13 (59.1)	9 (40.9)	0.83	-----
Tobacco consumers	78	48 (61.5)	30 (38.5)		

*Normal vs. HNSCC;

**Tobacco consumption habits include tobacco chewing (betel quid, areca nut or pan masala) and/or smoking of bidi or cigarettes.

The ability of nuclear PTMA to distinguish squamous cell hyperplasia, dysplasia and HNSCC from normal oral mucosa was determined by evaluating sensitivity, specificity, positive and negative predictive values and area-under-the-curve (AUC) using Receiver Operating Characteristic (ROC) curve analysis. The values for sensitivity, specificity and AUC for squamous cell hyperplasia, dysplasia and HNSCC are given in Table 5. The positive predictive values (PPV) were 86.0, 67.6 and 83.6 for nuclear expression of PTMA respectively in these three groups (Table 5).

**Table 5:** Biomarker analysis of PTMA in squamous cell hyperplasia, dysplasia and HNSCC.

PTMA	Sensitivity	Specificity	PPV	NPV	AUC
**1) Normal vs. Squamous cell hyperplasia**	63.8	88.0	86.0	67.7	0.764
**2) Normal vs. Dysplasia**	50.0	88.0	67.6	77.9	0.708
**3) Normal vs. HNSCC**	61.0	88.0	83.6	69.3	0.765

### PTMA overexpression as an independent prognostic marker for HNSCC

**Figure 2:**
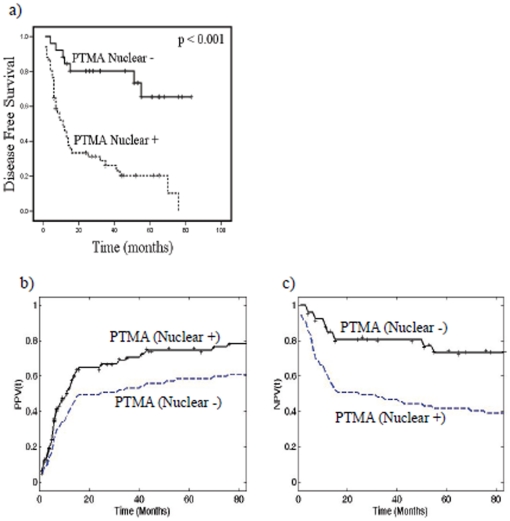
Survival analysis in HNSCC patients: (a) Kaplan–Meier estimation of cumulative proportion of disease-free survival, median time for disease-free survival (DFS: no recurrence/metastasis) in HNSCC patients showing nuclear immunostaining of PTMA was 11 months as compared to the patients showing no nuclear PTMA immunostaining (p<0.001); (**b**) **Positive Predictive Values:** Positive Predictive Values [PPV(t)] for time to cancer relapse for 51 HNSCC patients with PTMA(+) (solid line) and for all 77 HNSCC patients with survival data (dashed line); (**c**) **Negative Predictive Values:** Negative Predictive Values [NPV(t)] for time to cancer relapse for 26 patients with PTMA (−) (solid line), and for all 77 patients (dashed line).

The estimated predictive power of PTMA expression with poor prognosis was assessed by Kaplan-Meier survival analysis. HNSCC patients harboring nuclear PTMA showed significantly reduced disease-free survival (p<0.001; median survival 11 months) as compared to the patients showing no nuclear PTMA immunostaining (Figure 2d). Cox-multivariate regression analysis was carried out to determine the prognostic potential of nuclear PTMA in HNSCC as compared to other clincopathological parameters including age, gender, histological grade, T-stage, nodal status and tumor stage. Interestingly, nuclear PTMA emerged as an independent and most significant predictor of poor prognosis in HNSCC patients in multivariate analysis (p<0.001, HR = 5.2, 95% CI = 2.3–11.8).

Based on our data, the additional prognostic value that nuclear PTMA expression provided for predicting (PPV) or excluding (NPV) cancer recurrence in HNSCC patients was measured by the ratios: PPV_relapse/HNSCC_ (83 months | PTMA)/PPV_relapse/HNSCC_ (83 months) = 78.4/61.0; NPV_relapse/HNSCC_ (83 months | PTMA)/NPV_relapse/HNSCC_ (83 months) = 73.1/39.0 (Figure 2e & 2f). Hence, these findings clearly demonstrate the potential of nuclear PTMA as a marker for predicting recurrence in HNSCC.

### Verification of PTMA overexpression by RT-PCR and Immunoblotting

**Figure 3:**
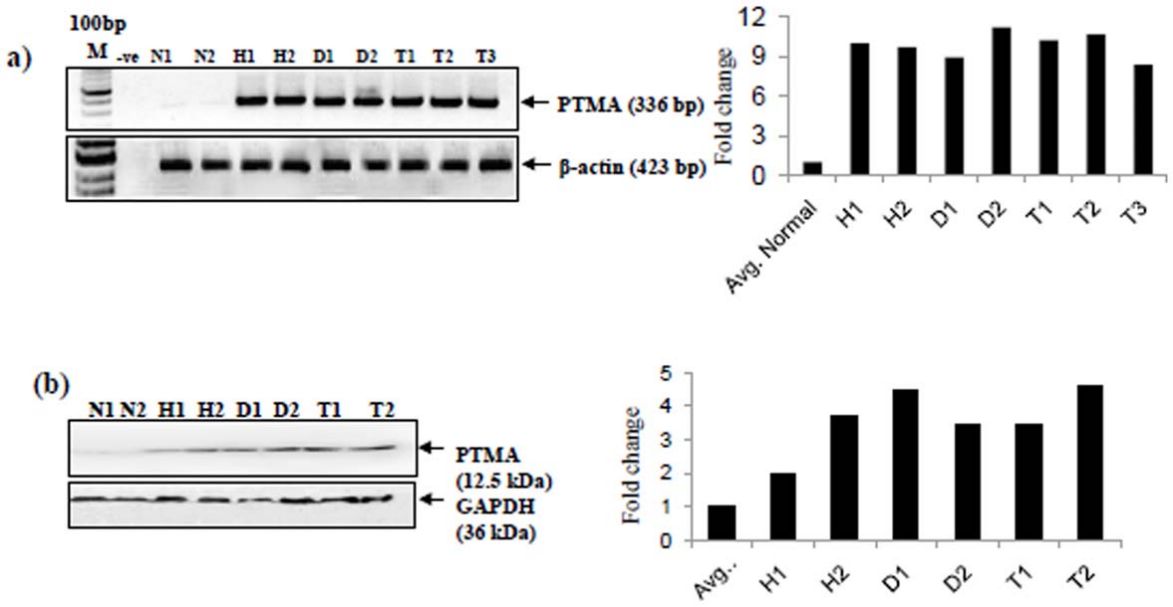
Verification of PTMA overexpression: (a) RT-PCR analysis of PTMA in normal mucosa, squamous cell hyperplasia, dysplasia and HNSCC tissues. Panel shows increased levels of PTMA transcripts in squamous cell hyperplasia (H1, H2), dysplasia (D1, D2) and HNSCC (T1, T2, T3) compared with the normal oral mucosa (N1, N2) that showed basal levels of PTMA transcripts. β-actin was used as a control to normalize the quantity of RNA used for each RT-PCR reaction is shown in the lower panel. (**b**) **Immunoblot analysis:** Immunoblot analysis of PTMA in normal oral mucosa (N1, N2), squamous cell hyperplasia (H1, H2), dysplasia (D1, D2) and HNSCC (T1, T2). Equal amount of protein lysates were electrophoresed on 12% SDS-PAGE and transferred to PVDF membrane. The membrane was incubated with respective primary antibodies and secondary antibodies as described in the Methods section and the signal detected by enhanced chemiluminescence method. Panel shows increased expression of PTMA in squamous cell hyperplasia (H1, H2), dysplasia (D1, D2) and HNSCC (T1, T2) compared with normal oral mucosa (N1, N2). GAPDH was used as loading control (lower panel).

The overexpression of PTMA in squamous cell hyperplasia, dysplasia and HNSCC in comparison to normal oral mucosa was verified by RT-PCR and immunoblotting in the same tissue samples as used for immunohistochemical analysis. Our RT-PCR analysis demonstrated increased levels of PTMA transcripts in squamous cell hyperplasia (H1, H2), dysplasia (D1, D2) and HNSCC (T1, T2, T3) in comparison with normal tissues (N1, N2) (Figure 3a). Immunoblot analysis using specific antibody for PTMA clearly showed a single intense band of 12.5 kDa in whole cell lysates obtained from squamous cell hyperplasia (H1, H2), dysplasia (D1, D2) and HNSCCs (T1, T2) while no band could be detected in normal oral mucosa (N1, N2) (Figure 3b). Together, these findings confirmed overexpression of PTMA in squamous cell hyperplasia, dysplasia and HNSCC in comparison to normal oral tissues.

## Discussion

Chronic inflammation in oral mucosa culminates in development of OPLs, some of which progress to frank malignancy. In this multistep model of head and neck tumorigenesis, oral lesions such as leukoplakia with histopathological evidence of dysplasia, also considered as pre-malignant or potentially malignant lesions, are at high risk of progression to cancer - often but not always correlating with the severity of epithelial dysplasia [31]. However, oral lesions with squamous cell hyperplasia have been largely excluded from molecular analysis in most of the studies. Herein, we demonstrated that alterations in protein expression may occur in early stages of head and neck carcinogenesis i.e. squamous cell hyperplasia, which might account for the increased proliferation. Another major clinical challenge is to evaluate the prognosis of HNSCC effectively, thereby emphasizing the urgent need for biomarkers that can predict the risk of recurrence in HNSCC. The salient findings of our study are: (i) significant increase in nuclear PTMA expression in squamous cell hyperplasia and dysplasia in comparison with normal oral tissues; (ii) the increased potential of nuclear PTMA to differentiate squamous cell hyperplasia, dysplasia and HNSCC from normal oral mucosa with high sensitivity and specificity; (iii) potential of nuclear PTMA in predicting recurrence in HNSCC. To the best of our knowledge, this investigation demonstrates the first clinical evidence of increased nuclear PTMA expression in pre-malignant lesions and HNSCC on large scale. Further, current study also verified our previous report of increased expression of PTMA protein in OPLs identified using iTRAQ labeling and tissue proteomic analysis, although in limited number of cases [32].

The significantly increased expression of nuclear PTMA in early lesions with squamous cell hyperplasia suggests its potential to identify patients at an early stage, before onset of dysplasia. This is well supported by the role of nuclear PTMA in regulation of cell cycle and proliferation [33]. Further, increased nuclear PTMA expression has also been reported in response to inflammation. Notably, thymosin alpha1 derived from its precursor PTMA has been reported to be an endogenous regulator of immune homeostasis that controls inflammation, immunity, and tolerance in a variety of clinical settings [34]. Importantly, chronic inflammation has been widely linked to the development of HNSCC [35] and our present findings of nuclear PTMA overexpression in squamous cell hyperplasia, dysplasia and frank tumors tempts us to speculate role of PTMA in linking inflammation and cancer development. We also recognize the possible link between nuclear PTMA expression and risk of cancer development needs confirmation in a long term follow-up study of patients with squamous cell hyperplasia; such longitudinal studies are challenging but these patients are being followed up for regular monitoring of disease progression.

In support of our findings, Suzuki et al., reported increased nuclear PTMA expression with progression from normal epithelium, through prostatic intraepithelial neoplasia to carcinomas, suggesting its involvement in differentiation and progression of prostate cancer [15]. Several other studies have also suggested a similar role for PTMA in cellular proliferation as an oncoprotein [5]–[7], [33], [36]–[38]. PTMA is not only related to proliferation, but is directly involved in the process, probably as an early component in the proliferation events triggered by the myc genes [8], [39]. It also affects the activity of specific transcription factors, including the estrogen receptor (ER), p53, p21 and signal transducers and activators of transcription 3 [36], [40], [41]. Notably, PTMA expression is positively regulated by estrogen RNA binding protein, HuR, c-myc and E2F, while p53 acts as a negative transcriptional regulator [40], [42]–[44]. Furthermore, PTMA interacts with histones and affects chromatin remodeling processes [36], [45]–[47]. In addition, the role of PTMA in adhesion, migration and proliferation has been reported in human ovarian cancer [48].

Most importantly, in our study nuclear PTMA expression emerged as an independent poor prognosticator in multivariate analysis, in comparison with the known clinical and pathological factors for HNSCC including histological grade [well differentiated (WD), moderately differentiated (MD) and poorly differentiated (PD)], T-stage, lymph node positivity and tumor stage. Further, analysis of the predictive potential of PTMA revealed its utility as a marker to identify aggressive HNSCC, supporting the association observed by Kaplan–Meier analysis and logistic regression analysis. Taken together, these findings demonstrated the potential of nuclear PTMA as a potential marker for predicting poor prognosis of HNSCC. However, biological role of PTMA in HNSCC progression and recurrence is not clearly understood, thus warranting further investigations in future studies. Altered expression profiling of PTMA has also been reported in several other malignancies [5], [9]–[12], [14]. Overexpression of PTMA has been reported in several neoplasms and the expression level is associated with prognosis, metastatic potential and overall survival of the patients. Its overexpression in breast cancer is associated with metastatic potential of tumor and with the risk of death [5]. It has been reported as a potential biomarker in colon cancer [49], a tumor marker for detection of transitional cell carcinoma of urinary bladder [16] and prognosticator in upper urinary tract transitional cell carcinoma [14].

In conclusion, nuclear PTMA was observed in majority of squamous cell hyperplasia dysplasia and HNSCC. Large-scale longitudinal studies are warranted for evaluating the potential of PTMA overexpression to identify patients with oral lesions at high risk of cancer development. Further, our study demonstrated significance of nuclear PTMA as poor prognosticator of HNSCC.

## Materials and Methods

### Patients and clinicopathological data collection, tissue specimens

The Institutional Human Ethics Committee of the All India Institute of Medical Sciences (AIIMS), New Delhi, India, approved this study prior to its commencement. A written informed consent approved by ethical committee was obtained from all participants involved in the study. Tissue specimens were obtained by diagnostic or therapeutic procedures from patients showing features of squamous cell hyperplasia (n = 116), or dysplasia (n = 50) attending the Outpatient Clinic of the Departments of Surgical Disciplines and Otorhinolaryngology, AIIMS, and from 100 HNSCC patients undergoing curative cancer surgery during the period 2002–2007, after obtaining the patients' consent. Wherever possible, non-malignant paired tissues (n = 43) were taken from a site distant from the surgically resected HNSCC. Non-malignant unpaired oral tissues (n = 57) were also collected from the patients attending the Outpatient Department of Dental Surgery for tooth extraction. Taken together, these 100 non-malignant oral tissues with histological evidence of normal epithelia constituted the normal group. After excision, tissues were immediately snap-frozen in liquid nitrogen and stored at −80°C in the Research Tissue Bank till further use; one part of the tissue was also collected in 10% formalin and embedded in paraffin for histopathological and immunohistochemical analysis. The histologically confirmed oral normal epithelia, oral lesions with squamous cell hyperplasia or with dysplasia, and HNSCC as revealed by H and E staining were used for immunohistochemistry as described earlier [17]. The patients' demographic, clinical, and pathological data were recorded in a pre-designed performa as described previously [17], [50]. The information documented included clinical TNM staging (tumor, node, metastasis based on the Union International Center le Cancer TNM classification of malignant tumors 2002), site of the lesion, histopathological grade (WD, MD, PD), age, gender, and tobacco consumption habits.

### Follow-up Study

Seventy-seven HNSCC patients who underwent treatment from 2002–2007 were investigated and evaluated in the head-and-neck cancer follow-up clinic at regular time intervals. Survival status of the HNSCC patients was verified and updated from the records of the Tumor Registry, Institute Rotary Cancer Hospital, AIIMS, as of December 2009. HNSCC patients were monitored for a maximum period of 83 months. As per the hospital protocol described earlier [50], [51], HNSCC patients with T_1_ and T_2_ tumors were treated with surgery alone, whereas the majority of patients with T_3_ and T_4_ diseases were treated by radical surgery followed by postoperative radical radiotherapy. The patients were revisited clinically on a regular basis and the time to recurrence was recorded. If a patient died, the survival time was censored at the time of death; the medical history, clinical examination, and radiological evaluation were used to determine whether the death had resulted from recurrent cancer (relapsing patients) or from any other causes. Disease-free survivors were defined as patients free from clinical and radiological evidence of local, regional, or distant relapse at the time of the last follow-up. Loco-regional relapse/death was observed in 51 of 77 (66%) patients monitored during the follow-up. Twenty six patients who did not show recurrence were alive until the end of follow-up period. Only disease-free survival (DFS) was evaluated in the present study, as the number of deaths due to disease progression did not allow a reliable statistical analysis. Disease-free survival was expressed as the number of months from the date of surgery to loco-regional relapse/death.

### Immunohistochemistry

Paraffin-embedded sections (5 µm) of human normal oral mucosa (n = 100), squamous cell hyperplasia (n = 116), dysplasia (n = 50) and HNSCC (n = 100) were collected on gelatin-coated slides. In brief, the sections were deparaffinized in xylene, hydrated in gradient alcohol, and pre-treated in a microwave oven for 10 min at 800 W and 5 min at 480 W in Tris - EDTA buffer (0.01 M, pH = 9.0) for antigen retrieval. The sections were incubated with hydrogen peroxide (0.3% v/v) in methanol for 30 min to quench the endogenous peroxidase activity, followed by blocking with 1% bovine serum albumin (BSA) to preclude non-specific binding. Thereafter, the slides were incubated with goat polyclonal anti-PTMA (N-18) antibody (4 µg/ml, sc-18205, Santa Cruz Biotechnology, CA) for 16 h at 4°C. The primary antibody was detected using the streptavidin-biotin complex with the Dako LSAB plus kit (Dako CYTOMATION, Glostrup, Denmark) and diaminobenzidine as the chromogen [32]. All procedures were carried out at room temperature unless otherwise specified. Slides were washed with Tris-buffered saline (TBS, 0.1 M, pH = 7.4), 3–5 times after each step. Finally, the sections were counterstained with Mayer's hematoxylin and mounted with D.P.X mountant. In the negative control tissue sections, the primary antibody was replaced by isotype specific non-immune mouse IgG. Tissue sections from bladder cancer were used as a positive control for PTMA expression [12]. The sections were evaluated by light microscopic examination using Olympus BX51 microscope.

### Evaluation of immunohistochemical staining

Each slide was evaluated for PTMA immunostaining using a semi-quantitative scoring system for both staining intensity and the percentage of positive epithelial cells as described earlier [50]. Immunopositive staining was evaluated in randomly selected five areas of the tissue section. For PTMA protein expression, sections were scored as positive, if epithelial cells showed immunopositivity in the nucleus when observed independently by four of us (SCT, AM, JK, SDG), who were blinded to the clinical outcome (the slides were coded and the scorers did not have prior knowledge of the local tumor burden, lymphonodular spread, and grading of the tissue samples). The tissue sections were scored based on the % of immunostained cells as: 0–10% = 0; 10–30% = 1; 30–50% = 2; 50–70% = 3 and 70–100% = 4. Sections were also scored semi-quantitatively on the basis of staining intensity as negative = 0; mild = 1; moderate = 2; intense = 3 [50]. Finally, a total score was obtained by adding the score of percentage positivity and intensity. The scoring by all four observers (SCT, AM, JK, SDG) was discrepant in about 5% cases and a consensus on the final result was reached by re-evaluation of these slides and discussion.

### Statistical Analysis

The immunohistochemical data were subjected to statistical analysis using the SPSS 13.0 software (Chicago). Sensitivity and specificity were calculated and quantified using receiver operating characteristic (ROC) curve analysis. The predictive value (PV) describes the proportion of correctly classified cases. Based on sensitivity and specificity values for PTMA, a cut-off ≥2 was defined as positive criterion for nuclear PTMA immunopositivity for statistical analysis. The relationships between PTMA protein expression and clinicopathological parameters were tested using Chi-Square and Fischer's exact test. Two-sided p values were calculated and p<0.05 was considered to be significant. Similarly, positive predictive value (PPV) and negative predictive value (NPV) was calculated for squamous cell hyperplasia, dysplasia and HNSCC with respect to normal tissues.

The correlation of PTMA staining with patient survival was evaluated using life tables constructed from survival data with Kaplan-Meier plots [50]. Multivariate analysis was carried out using Cox regression model which included PTMA overexpression and clinicopathological parameters such as age, gender, histological grade, T-stage, nodal status and tumor stage. The systematic and rigorous assessment of Positive and Negative Predictive Values (PPV and NPV respectively) for prognostic biomarkers was carried out as described earlier [50]. For the follow-up study of HNSCC, let T denote the failure time, i.e., the first time recurrence is diagnosed after surgical removal of the tumor. For these data, the positive and negative predictive values as functions of time are defined as follows: PPV_tumor_(t) = Prob (T≤t and Recurrence| PTMA (nuclear)≥2); NPV_tumor_(t) = Prob (T>t OR No Recurrence| PTMA (nuclear)<2); 0≤t≤83, These probabilities are estimated from the observed accumulated incidences over the respective time periods [52].

### Reverse Transcription-PCR

Representative frozen tissue specimens of histologically confirmed oral normal tissues (n = 5), squamous cell hyperplasia (n = 5), dysplasia (n = 5) and HNSCC (n = 5) were used for extraction of total RNA using the TRI reagent (Sigma, MO) as previously described [53]. First-strand cDNA was synthesized using 2 µg RNA with oligo dT as the primer with MMLV reverse transcriptase. PCR was carried out using PTMA specific primers forward (5′-ATGTCAGACGCAGCCGTAGACACCA-3′) and reverse (5′-CTAGTCATCCTCGTCGGTCTTCTGC-3′) [11]. Twenty microliters of each PCR product was used for electrophoresis on a 1.2% agarose gel and photographed using ChemiImager system.

### Immunoblot analysis of PTMA in oral tissues

Whole-cell lysates were prepared from oral normal mucosa (n = 5), squamous cell hyperplasia (n = 5), dysplasia (n = 5), and HNSCC (n = 5) tissues by homogenization in lysis buffer containing 50 mM Tris-Cl (pH = 7.5), 150 mM NaCl, 10 mM MgCl_2_, 1 mM ethylenediamine tetraacetate (EDTA, pH = 8.0), 1% Nonidet P-40, 1 mM phenylmethylene sulfonylfluoride (PMSF) and 2 µl/ml protease inhibitor cocktail (Sigma) as previously described [17], [32]. Protein concentration was determined using the Bradford reagent (Sigma) and equal amounts of proteins (50 µg/lane) were resolved on 12% sodium dodecyl sulfate (SDS)-polyacrylamide gel. The proteins were then electro-transferred onto polyvinylidene-difluoride (PVDF) membrane. After blocking with 5% non-fat powdered milk in Tris-buffered saline (TBS, 0.1 M, pH = 7.4), blots were incubated with polyclonal anti-PTMA antibody (2 µg/ml, sc-18205, Santa Cruz Biotechnology, CA) at 4°C overnight. Protein abundance of GAPDH (mouse monoclonal antibody, Abcam Inc., Cambridge, MA) served as a control for protein loading in each lane. Membranes were incubated with their respective HRP-conjugated secondary antibody, (DAKO Cytomation, Glostrup, Denmark), diluted at an appropriate dilution in 1% BSA, for 2 h at room temperature. After each step, blots were washed three times with Tween (0.1%)-Tris-buffer saline (TTBS). Protein bands were detected by the enhanced chemiluminescence method (ECL, Pierce, IL) on XO-MAT film.
